# Changes in Calorie Content of Menu Items at Large Chain Restaurants After Implementation of Calorie Labels

**DOI:** 10.1001/jamanetworkopen.2021.41353

**Published:** 2021-12-30

**Authors:** Anna H. Grummon, Joshua Petimar, Mark J. Soto, Sara N. Bleich, Denise Simon, Lauren P. Cleveland, Anjali Rao, Jason P. Block

**Affiliations:** 1 Department of Nutrition, Harvard T.H. Chan School of Public Health, Boston, Massachusetts; 2 Department of Population Medicine, Harvard Medical School, Harvard Pilgrim Health Care Institute, Boston, Massachusetts; 3 Department of Health Policy and Management, Harvard T.H. Chan School of Public Health, Boston, Massachusetts

## Abstract

**Importance:**

Calorie labeling on menus is required in US chain food establishments with 20 or more locations. This policy may encourage retailers to offer lower-calorie items, which could lead to a public health benefit by reducing customers’ calorie intake from prepared foods. However, potential reformulation of restaurant menu items has not been examined since nationwide enforcement of this policy in 2018.

**Objective:**

To examine the calorie content of menu items at large chain restaurants before and after implementation of federally mandated menu calorie labels.

**Design, Setting, and Participants:**

This pre-post cohort study used restaurant menu data from MenuStat, a database of nutrition information for menu items offered in the largest chain restaurants in the US, collected annually from 2012 to 2019. The study comprised 35 354 menu items sold at 59 large chain restaurants in the US. Statistical analysis was conducted from February 4 to October 8, 2021.

**Intervention:**

Nationwide implementation of menu calorie labeling.

**Main Outcomes and Measures:**

Changes in menu items’ calorie content after restaurant chains implemented calorie labels were estimated, adjusting for prelabeling trends. All menu items, continuously available items, items newly introduced to menus, and items removed from menus were examined separately.

**Results:**

Among the 59 restaurant chains included in the study, after labeling, there were no changes in mean calorie content for all menu items (change = −2.0 calories; 95% CI, −8.5 to 4.4 calories) or continuously available items (change = −2.3 calories; 95% CI, −11.5 to 6.3 calories). Items that were newly introduced after labeling, however, had a lower mean calorie content than items introduced before labeling (change = −112.9 calories; 95% CI, −208.6 to −25.2 calories), although there was heterogeneity by restaurant type. Items removed from menus after labeling had similar calorie content as items removed before labeling (change = 0.5 calories; 95% CI, −79.4 to 84.0 calories).

**Conclusions and Relevance:**

In this cohort study of large chain restaurants, implementing calorie labels on menus was associated with the introduction of lower-calorie items but no changes in continuously available or removed items.

## Key Points

**Question:**

Is calorie labeling on menus associated with reductions in the calorie content of menu items at large chain restaurants?

**Findings:**

This cohort study comprising 59 large restaurant chains followed up from 2012 to 2019 found that restaurants did not change the calorie content of continuously offered items. However, new items introduced after calorie labeling had a mean of 113 fewer calories (approximately −25%) compared with new items introduced before labeling, a statistically significant reduction.

**Meaning:**

This study suggests that mandated calorie labeling may have encouraged large restaurant chains to introduce lower-calorie items, but additional interventions should be explored to improve the nutritional quality of foods purchased from restaurants.

## Teaser

This cohort study uses data from the MenuStat database to examine the calorie content of menu items at large chain restaurants before and after implementation of federally mandated menu calorie labels.

## Introduction

The calorie menu labeling provision of the 2010 Patient Protection and Affordable Care Act (P.L. 111-148) requires food establishments with 20 or more US locations to post the calorie content of prepared foods on menus alongside the item’s price.^1^ The goals of the policy, enforced as of May 2018, are to encourage customers to purchase lower-calorie foods (change what consumers demand) and to prompt retailers to offer lower-calorie options (change what restaurants supply). Most evaluations of this policy have examined changes in consumer behavior,^2,3,4,5,6,7,8^ with the studies with the largest samples finding 4% to 6% reductions in calories purchased after labeling.^7,8^

Fewer studies have examined retailer reformulation after calorie labeling. Calorie labeling policies could prompt restaurants to reduce the calorie content of menu items, for example, by reformulating existing items to have fewer calories, introducing lower-calorie items, or removing high-calorie items from their menus. These changes could appreciably reduce calorie consumption by default, even without behavior change from customers, given that prepared foods from restaurants and other sources outside the home account for one-third of US individuals’ daily calorie intake.^9^ Several studies conducted before nationwide implementation of calorie labeling identified small declines in calorie content of restaurant offerings since 2012, particularly for newly introduced items.^10,11,12,13,14^ Although some restaurants in those studies were subject to local calorie labeling regulations, national chains may have waited until the nationwide rollout of calorie labels to consider reformulation rather than respond to local policies that affected only some restaurants.

Understanding the degree to which restaurants reformulated menu items after the nationwide implementation of calorie labeling is important for estimating the long-term health and economic outcomes associated with this policy.^15^ This study aimed to evaluate changes in the calorie content of menu items offered at 59 of the top revenue-generating restaurant chains in the US after implementation of national calorie labeling.

## Methods

### Design and Setting

We used a pre-post design to examine changes in the calorie content of menu items at chain restaurants after implementation of federally mandated menu calorie labels. Enforcement of the mandate was delayed several times.^16^ The last delay came on May 4, 2017, 1 day before the scheduled enforcement date, which resulted in a final enforcement date of May 7, 2018.^16^ Audit studies indicated that many chains implemented labeling by the 2017 enforcement date, and nearly all of the remainder implemented labels in 2018.^17,18^ This study did not meet the definition of human subjects research per federal regulations under the purview of the Harvard Pilgrim Health Care Institute institutional review board. This study followed the Strengthening the Reporting of Observational Studies in Epidemiology (STROBE) reporting guideline.

### Data Source

Data for this study came from MenuStat, a database developed by the New York City Department of Health and Mental Hygiene (NYC DOHMH) to track the nutritional content of items sold at the approximately 100 top-selling US restaurant chains.^19^ Details on MenuStat data collection are available elsewhere.^20^ In brief, in January of each year, MenuStat retrieved nutrition information about menu items from restaurants’ public websites and categorized items into 1 of 12 mutually exclusive menu categories (eg, entrees, desserts). The NYC DOHMH did not release a MenuStat database in 2019, so the research team created our own database using the same methods and guidance from NYC DOHMH^20^ (eMethods in the Supplement).

We restricted analyses to the 66 chains included in all years of MenuStat between 2012 (the first year of annual MenuStat data) and 2019 (the most recent year currently available) (eFigure in the Supplement). We excluded 5 chains that implemented calorie labeling in 2013 or earlier (for which we would not have sufficient prelabeling data) and 2 that were noncompliant with labeling by the 2018 enforcement date, yielding an analytic sample of 59 chains. Each year of data included a record of all menu items sold at each chain (133 920 item-by-year observations). For each chain, we excluded data from the year in which the chain implemented labeling because we could not ascertain whether calorie labels were implemented before or after MenuStat collected that year’s nutrition data.

### Measures

#### Calorie Labeling Period

Data on the timing of calorie labeling at each chain came from 2 audits of top restaurant chains conducted by Cleveland and colleagues.^17,18^ The first study examined 90 chains (including all 59 chains analyzed in our study) from May to November 2017 and found that 71 chains (47 of those in our sample) had implemented calorie labeling either regionally or nationally at the time of the audit^17^ (these chains likely moved forward with implementation in 2017 given the last-minute delay in enforcement date). We coded these chains as implementing labeling in 2017 and considered 2012 to 2016 the prelabeling period for these 47 chains. A second audit in June to December 2018, after the final enforcement date of May 2018, found that 94% of the restaurant chains examined had implemented calorie labeling.^18^ We coded the 12 chains in our sample that had implemented calorie labels by the second audit, but not the first, as implementing menu calorie labeling in 2018, and considered 2012 to 2017 the prelabeling period for these 12 chains.

#### Outcomes

Our primary outcome was calorie content of menu items (calories per item) as recorded in MenuStat. When an item was missing calorie information in a particular year, we replaced those missing values (1001 unique items) with the item’s calorie value from the nearest year available within the same labeling period.

We examined potential changes in calorie content of menu items in several ways. First, we examined calorie content of all menu items offered to provide an overall assessment of foods and beverages available to consumers at top chain restaurants. Second, we assessed continuously available menus items to shed light on potential reformulation of existing items. Continuously available menu items were defined as items offered by chains that had calorie information every year from 2012 to 2019 (identified by matching on restaurant name, item name, menu description, and item category). Third, we assessed items newly introduced to menus, defined as those introduced after 2012. A menu item was considered newly introduced only once, during the year in which it first became available. Because the study period began in 2012, we could not determine items that were new in 2012; analyses of new items examine data from 2013 to 2019. Fourth, we assessed items removed from menus, defined as those offered between 2012 and 2018 and removed (ie, no longer on the menu) between 2013 and 2019. We evaluated removed items in the first year that they no longer appeared on the menu.

We examined menu items overall as well as by menu item type and restaurant type (eTable 1 in the Supplement). For menu item type, we used MenuStat’s categorization system^19^ and examined entrees (eg, burgers), appetizers and sides (eg, French fries), desserts (eg, cookies), toppings and ingredients (eg, salad dressing), all foods excluding toppings and ingredients (ie, entrees, appetizers and sides, and desserts), and beverages. For restaurant type, we used the categorization system of Bleich et al^21^ and examined fast-food restaurants, fast casual restaurants, full service restaurants, and coffee shops.

### Statistical Analysis

Statistical analysis was conducted from February 4 to October 8, 2021. The analytic sample excluded items with missing calorie information that could not be imputed from another year of MenuStat data (7062 of 42 416 unique menu items [17%]; eFigure in the Supplement). The rate of missingness was slightly higher in the postlabeling period than the prelabeling period overall (4972 of 36 205 [14%] vs 9330 of 80 979 [12%]) and for removed items (3230 of 9933 [33%] vs 2513 of 14 406 [17%]), but was lower in the postlabeling period for new items (1905 of 9522 [20%] vs 4219 of 16 553 [25%]) (eTables 2 and 3 in the Supplement). The composition of menu item types and restaurant types, however, was similar among observations analyzed in the prelabeling vs postlabeling period (eTable 4 in the Supplement) and for items with calorie information compared with the overall sample (eTable 5 in the Supplement). The final analytic sample included 102 882 item-by-year observations from 35 354 unique items. eTable 6 in the Supplement provides observation counts by labeling period, menu item type, and restaurant type.

Primary analyses estimated the association of calorie labeling implementation with mean calorie content of menu items using a pre-post design, adjusting for potential secular trends. All analyses regressed calories per item on labeling period (before vs after) and years since study start in 2012 (eMethods in the Supplement provide additional details). All analyses estimated robust SEs clustered at the chain level.^22,23^

We conducted 7 sensitivity analyses. First, we examined outcomes at the median instead of the mean, using quantile regression. Second, we analyzed outcomes without imputing calorie content for any items. Third, we excluded 4 restaurant chains for which more than 50% of locations were subject to local calorie labeling regulations prior to 2012, as these chains may have had incentive to reformulate menu items prior to the study start. We assessed the proportion of restaurant locations subject to local regulations using publicly available data on counties subject to local regulations^24^ merged with restaurant location data purchased from AggData.com.^25^ Fourth, we recoded the 9 chains that had regionally (but not nationally) implemented calorie labels in 2017 as implementing calorie labeling in 2018 instead of 2017, to assess changes in calorie content after all chains had implemented labeling nationwide. Fifth, to assess results among early compliers, we included only chains that implemented labeling by 2017, excluding those that were not compliant until 2018. Sixth, to assess results among nationally available items, we excluded items available only in certain regions. Seventh, to assess outcomes among non–limited-run newly introduced items, we analyzed only new items that remain on menus for at least 1 year after they were introduced.

To provide insight on whether calorie labeling was associated with changes in the calorie content of higher-calorie items, secondary analyses examined changes in outcomes at the 90th percentile using quantile regressions with a similar pre-post analytic approach as in the primary analyses.^26^

Analyses calculated 2-sided 95% CIs and accounted for multiple comparisons within families of outcomes (ie, all menu items [1 test], menu item categories [6 tests], and restaurant type categories [4 tetsts]) by controlling the false discovery rate at *q* = 0.05 using the Benjamini-Hochberg linear step-up method.^27^ Adjusted *P* < .05 was considered statistically significant. Analyses were conducted in Stata IC, version 15.1 (StataCorp LLC).

## Results

In the prelabeling period, the average menu item offered at the 59 restaurant chains contained a mean (SD) of 399 (382) calories (Table 1). The prelabeling mean (SD) calorie content was 369 (330) for continuously available items, 457 (423) for newly introduced items, and 478 (396) for removed items.

**Table 1.** Unadjusted Calories of Menu Items Sold in Chain Restaurants Before and After Implementation of Menu Calorie Labels^a^

Category	Calories, mean (SD)							
	All items offered from 2012 to 2019^b^		Items offered every year from 2012 to 2019^c^		Items newly introduced in 2013 to 2019^d^		Items removed in 2013 to 2019^e^	
	Before	After	Before	After	Before	After	Before	After
All menu items	399 (382)	388 (398)	369 (330)	373 (319)	457 (423)	376 (415)	478 (396)	375 (377)
By menu item type								
Food^f^	530 (409)	535 (448)	470 (337)	474 (319)	592 (443)	584 (502)	603 (394)	563 (413)
Entrees^g^	583 (403)	560 (386)	528 (340)	528 (320)	628 (399)	585 (409)	650 (387)	597 (368)
Appetizers and sides^h^	384 (381)	450 (536)	344 (277)	352 (279)	449 (504)	562 (673)	411 (391)	444 (455)
Desserts	514 (425)	556 (564)	442 (348)	452 (321)	611 (548)	623 (749)	551 (361)	584 (578)
Toppings and ingredients	113 (128)	110 (130)	87 (92)	86 (87)	143 (159)	106 (134)	127 (142)	120 (157)
Beverages	294 (289)	279 (268)	272 (270)	279 (272)	356 (358)	265 (231)	355 (330)	315 (302)
By restaurant type								
Fast food	393 (382)	371 (397)	363 (290)	366 (287)	467 (429)	332 (423)	476 (381)	327 (357)
Fast casual	311 (350)	339 (370)	375 (369)	352 (351)	221 (238)	339 (338)	314 (341)	304 (329)
Full service	457 (414)	451 (436)	396 (387)	422 (379)	539 (464)	458 (456)	553 (439)	466 (435)
Coffee	276 (171)	292 (196)	254 (171)	242 (171)	288 (175)	307 (214)	300 (162)	288 (182)

^a^ Data are from 35 354 menu items with calorie information offered at 59 restaurants in the MenuStat database from 2012 to 2019.

^b^ Offered on any menu between 2012 and 2019.

^c^ Items with the same name and description offered by the chain every year from 2012 to 2019 with calorie information available in all years.

^d^ Not offered in 2012 and introduced after 2012. Calories are measured in year of introduction.

^e^ Offered in 2012 to 2018 and removed between 2013 and 2019. Calories are measured in the year item was removed from menu (eg, items on the menu in 2015 and not on the menu in 2016 are measured in 2016).

^f^ Includes all food categories except toppings and ingredients.

^g^ Includes burgers, entrees, pizza, salad, sandwiches, and soup.

^h^ Includes appetizers and sides, baked goods, and fried potatoes.

When examining all menu items together, calorie content of menu items did not change after labeling overall (change = −2.0 calories; 95% CI, −8.5 to 4.4 calories) or by menu item type or restaurant type (Table 2^27^; Figure). Likewise, calorie content of items that were continuously offered did not change after labeling overall (change = −2.3 calories; 95% CI, −11.5 to 6.3 calories) or among specific menu items or restaurant types.

**Table 2.** Adjusted Changes in Mean Calorie Content of Menu Items After Implementation of Calorie Labeling^a^

Category	All items offered from 2012 to 2019^b^		Items offered every year from 2012 to 2019^c^		Items newly introduced in 2013 to 2019^d^		Items removed in 2013 to 2019^e^	
	Mean change in calories (95% CI)	Adjusted *P* value	Mean change in calories (95% CI)	Adjusted *P* value	Mean change in calories (95% CI)	Adjusted *P* value	Mean change in calories (95% CI)	Adjusted *P* value
All menu items	−2.0 (−8.5 to 4.4)	.54	−2.3 (−11.5 to 6.3)	.70	−112.9 (−208.6 to −25.2)	.009^f^	0.5 (−79.4 to 84.0)	.99
By menu item type								
Food^g^	−4.6 (−15.4 to 6.3)	.82	−7.8 (−21.6 to 5.3)	.54	−41.5 (−192.8 to 91.3)	.79	25.8 (−64.0 to 113.4)	.84
Entrees^h^	−7.3 (−23.1 to 8.6)	.82	−14.3 (−38.5 to 6.6)	.54	−24.3 (−124.3 to 79.7)	.79	37.5 (−58.1 to 118.5)	.84
Appetizers and sides^i^	−1.5 (−13.4 to 10.5)	.91	−1.2 (−16.1 to 13.3)	.88	−113.3 (−518.3 to 169.8)	.90	10.6 (−98.1 to 117.8)	.84
Desserts	−0.7 (−12.8 to 11.3)	.91	6.8 (−14.8 to 30.1)	.54	−84.3 (−287.3 to 122.8)	.69	53.2 (−100.8 to 222.1)	.84
Toppings and ingredients	2.7 (−1.9 to 7.3)	.82	5.6 (−3.8 to 19.7)	.54	−70.1 (−152.4 to 13.8)	.69	26.8 (−73.7 to 114.7)	.84
Beverages	1.9 (−4.8 to 8.7)	.87	7.2 (−5.3 to 23.1)	.54	−71.8 (−234.1 to 46.9)	.69	−85.9 (−198.0 to 57.8)	.84
By restaurant type								
Fast food	4.7 (−0.8 to 10.2)	.27	5.8 (−1.3 to 12.8)	.38	−181.2 (−369.9 to −18.2)	.07	−58.4 (−224.3 to 82.3)	.47
Fast casual	1.7 (−11.6 to 15.0)	.81	0.5 (−10.0 to 5.2)	.83	180.1 (24.7 to 445.1)	<.001^f^	178.3 (76.5 to 353.7)	<.001^f^
Full service	−12.1 (−28.1 to 3.8)	.27	−16.2 (−46.7 to 5.0)	.38	−98.0 (−235.0 to 16.0)	.13	56.3 (−90.4 to 190.9)	.47
Coffee	−1.2 (−7.7 to 5.3)	.81	2.9 (−23.4 to 16.9)	.83	−59.9 (−201.1 to 17.6)	.25	−72.1 (−207.2 to 55.9)	.47

^a^ Data are from 35 354 menu items with calorie information offered at 59 restaurants in the MenuStat database from 2012 to 2019 and show unstandardized regression coefficients and 95% CIs for estimated change in mean calorie content before and after implementation of menu calorie labels, adjusted for year (continuous). *P* values were adjusted within families of outcomes (ie, all menu items [1 test], menu item categories [6 tests], and restaurant type categories [4 tests]) by controlling the false discovery rate at *q* = 0.05 using the Benjamini-Hochberg linear step-up method.^27^

^b^ Offered on any menu between 2012 and 2019.

^c^ Items with the same or nearly identical name and description offered by the chain every year from 2012 to 2019 with calorie information available in all years.

^d^ Not offered in 2012 and introduced after 2012.

^e^ Offered in 2012 to 2018 and removed between 2013 and 2019.

^f^ Statistically significant at *P* < .05.

^g^ Includes all food categories except toppings and ingredients.

^h^ Includes burgers, entrees, pizza, salad, sandwiches, and soup.

^i^ Includes appetizers and sides, baked goods, and fried potatoes.

**Figure.**
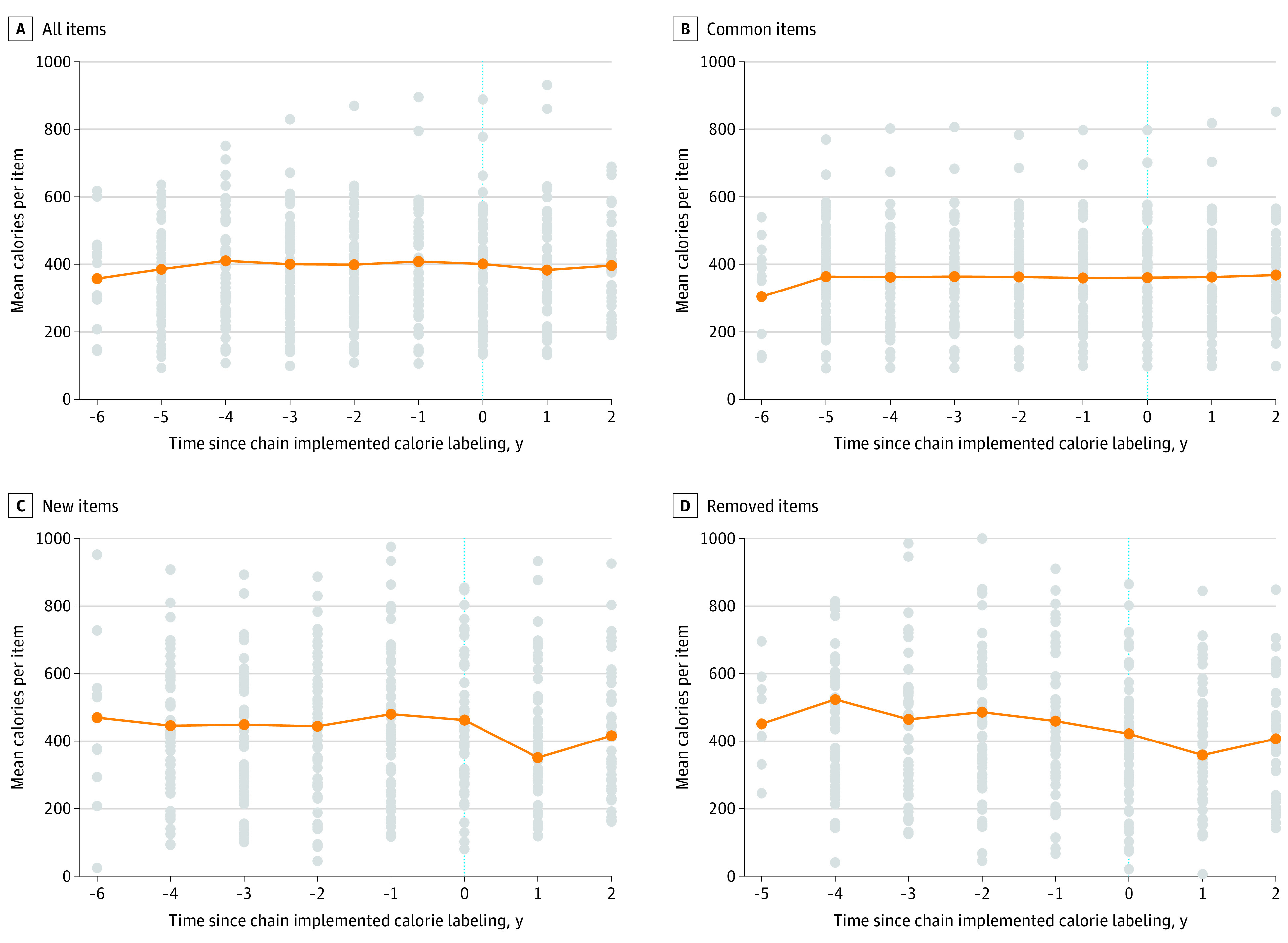
Mean Calorie Content of Menu Items Over Time A, All menu items. B, Common menu items. C, New menu items. D, Removed menu items. The graphs show the mean calories per item before and after menu calorie labeling. The mean calories per item for each individual restaurant are indicated by the gray dots, the mean calories per item across all restaurants are indicated by the orange dots, and the trend over time is represented by the orange line. The vertical dotted line indicates the time of implementation of labeling.

Items that were newly introduced after labeling had a mean of 112.9 fewer calories (95% CI, −208.6 to −25.2 calories) than items introduced before labeling, an approximate 25% decrease (Table 2; Figure). This decrease was observed especially among fast-food chains (change = −181.2 calories; 95% CI, −369.9 to −18.2 calories). Full-service and coffee restaurant chains showed relatively smaller decreases with 95% CIs that crossed 0 (full-service chains: change = −98.0 calories; 95% CI, –235.0 to 16.0 calories; coffee chains: change = −59.9 calories; 95% CI, –201.1 to 17.6 calories). Conversely, in fast casual restaurant chains, new items had a higher calorie content after labeling (change = 180.1 calories; 95% CI, 24.7-445.1 calories). Post hoc exploratory analyses of macronutrient content (ie, saturated and unsaturated fat, sugar, nonsugar carbohydrates excluding fiber, and protein) among newly introduced items found reductions for most nutrients after labeling (eTable 7 in the Supplement).

Overall, items removed from menus after labeling did not have different calorie content than those removed before labeling (change = 0.5 calories; 95% CI, −79.4 to 84.0 calories) (Table 2; Figure), perhaps because of an existing decrease in calorie content of removed items (trend in calorie content for removed items = −17 calories/year). However, menu items removed by fast casual restaurants after labeling had higher calorie contents than items removed by these restaurants before labeling (change = 178.3 calories; 95% CI, 76.5-353.7 calories).

Sensitivity analyses revealed a similar pattern of results (eTable 8 in the Supplement). One exception was that the reduction in calorie content among newly introduced items was smaller when excluding limited-run items (change = −61.9 calories; 95% CI, −201.5 to 68.9 calories).

Secondary analyses of higher-calorie items found that, among all menu items, the 90th percentile of calorie content increased slightly after labeling (change = 7.5 calories; 95% CI, 5.1-9.9 calories) (Table 3).^27^ Similarly, the 90th percentile of calorie content increased among continuously available items after labeling (change = 19.1 calories; 95% CI, 15.3-23.0 calories). These increases were observed in most menu item types and restaurant types. By contrast, the 90th percentile of newly introduced items was lower after labeling than before (change = −184.7 calories; 95% CI, −255.1 to −114.3 calories), with the largest decreases observed among fast-food restaurant chains (change = −401.3 calories; 95% CI, –539.0 to –263.6 calories) and full-service restaurant chains (change = −96.4 calories; 95% CI, –185.0 to –7.8 calories). The 90th percentile of calorie content was similar for items removed after labeling compared with those removed before labeling (change = 10.7 calories; 95% CI, −33.1 to 54.5 calories), with heterogeneity by menu item and restaurant type.

**Table 3.** Adjusted Changes in Calorie Content at the 90th Percentile After Implementation of Calorie Labeling^a^

Category	All items offered from 2012 to 2019^b^		Items offered every year from 2012 to 2019^c^		Items newly introduced in 2013 to 2019^d^		Items removed in 2013 to 2019^e^	
	Mean change in calories (95% CI)	Adjusted *P* value	Mean change in calories (95% CI)	Adjusted *P* value	Mean change in calories (95% CI)	Adjusted *P* value	Mean change in calories (95% CI)	Adjusted *P* value
All menu items	7.5 (5.1 to 9.9)	<.001^f^	19.1 (15.3 to 23.0)	<.001^f^	−184.7 (−255.1 to −114.3)	<.001^f^	10.7 (−33.1 to 54.5)	.63
By menu item type								
Food^g^	9.5 (6.3 to 12.7)	<.001^f^	21.6 (16.8 to 26.5)	<.001^f^	−94.1 (−201.0 to 12.8)	.17	59.7 (4.1 to 115.2)	.07
Entrees^h^	11.9 (6.5 to 17.4)	<.001^f^	22.5 (13.9 to 31.0)	<.001^f^	−8.2 (−82.9 to 66.6)	≥.99	66.3 (10.1 to 122.5)	.06
Appetizers and sides^i^	10.3 (3.0 to 17.7)	.007^f^	24.0 (13.3 to 34.7)	<.001^f^	0.6 (−255.5 to 256.8)	≥.99	9.1 (−124.2 to 142.5)	.89
Desserts	15.4 (−2.7 to 33.6)	.10	30.7 (−1.0 to 62.3)	.06	−22.3 (−499.8 to 455.2)	≥.99	130.3 (−147.2 to 407.8)	.43
Toppings and ingredients	6.5 (3.8 to 9.2)	<.001^f^	16.2 (11.6 to 20.8)	<.001^f^	−123.2 (−194.1 to −52.3)	.002^f^	47.5 (−7.8 to 102.8)	.14
Beverages	6.0 (2.2 to 9.9)	.003^f^	18.7 (12.4 to 25.0)	<.001^f^	−153.2 (−230.0 to −76.4)	.001^f^	−143.7 (−221.7 to −65.8)	.002^f^
By restaurant type								
Fast food	11.6 (8.6 to 14.5)	<.001^f^	22.2 (18.1 to 26.4)	<.001^f^	−401.3 (−539.0 to −263.6)	<.001^f^	−122.0 (−195.5 to −48.5)	.002^f^
Fast casual	19.5 (9.7 to 29.4)	<.001^f^	35.5 (22.3 to 48.7)	<.001^f^	392.8 (212.5 to 573.0)	<.001^f^	444.3 (229.6 to 659.1)	<.001^f^
Full service	12.3 (2.2 to 22.4)	.02^f^	19.9 (6.4 to 33.4)	.005^f^	−96.4 (−185.0 to −7.8)	.04^f^	116.8 (41.1 to 192.4)	.003^f^
Coffee	2.1 (−0.9 to 5.2)	.17	7.3 (1.6 to 13.1)	.01^j^	−32.1 (−93.3 to 29.2)	.31	−87.1 (−146.4 to −27.8)	.004^f^

^a^ Data are from 35 354 menu items with calorie information offered at 59 restaurants in the MenuStat database from 2012 to 2019 and show unstandardized regression coefficients and 95% CIs for estimated change in the 90th percentile of calorie content before and after implementation of menu calorie labels, adjusted for year (continuous). *P* values were adjusted within families of outcomes (ie, all menu items [1 test], menu item categories [6 tests], and restaurant type categories [4 tests]) by controlling the false discovery rate at *q* = 0.05 using the Benjamini-Hochberg linear step-up method.^27^

^b^ Offered on any menu between 2012 and 2019.

^c^ Items with the same or nearly identical name and description offered by the chain every year from 2012 to 2019 with calorie information available in all years.

^d^ Not offered in 2012 and introduced after 2012.

^e^ Offered in 2012 to 2019 and removed between 2013 and 2019.

^f^ Statistically significant at *P* < .05.

^g^ Includes all food categories except toppings and ingredients.

^h^ Includes burgers, entrees, pizza, salad, sandwiches, and soup.

^i^ Includes appetizers and sides, baked goods, and fried potatoes.

## Discussion

In this cohort study of large restaurant chains, we did not observe changes in the calorie content of all menu items after implementation of calorie labels. There were no differences in the calorie content of continuously available items before vs after labeling, or in items removed from menus after adjusting for prelabeling trends. By contrast, analyses revealed that menu items newly introduced after labeling contained approximately 25% fewer calories than menu items introduced before labeling. This decrease in calorie content of newly introduced items (which represent approximately one-fifth of all menu items in any given year) could lead to reductions in calories purchased or consumed if customers purchased these items in place of higher-calorie options. A meta-analysis of 26 studies found beneficial changes in sodium and fiber consumption after food and beverage reformulation initiatives, suggesting that reformulation may be associated with improvements in dietary intake.^28^ More than 30% of US individuals’ daily calorie intake comes from restaurant meals and other prepared foods^9^; the large contribution of these foods to US individuals’ daily diets suggests that even small proportional reductions in prepared food intake owing to calorie labeling could yield population health benefits.^15^

The decrease in calorie content of newly introduced items was observed both at the mean (approximately 113 fewer calories, a 25% decrease) and among higher-calorie offerings (approximately 185 fewer calories, an 18% decrease, for items at the 90th percentile of calorie content), although decreases were smaller when excluding limited-run products. The overall decreases in calorie content observed among newly introduced items are consistent with and even larger than several earlier studies of chain restaurants, which found reductions in calorie content of newly introduced items from 2012 to 2015,^10,12^ especially in restaurants that voluntarily implemented calorie labeling.^21^ A study of 2 supermarket chains also documented a decrease in the calorie content of newly introduced prepared bakery items after implementation of calorie labeling.^29^ Our study, to our knowledge the first to examine chain restaurants after nationwide implementation of menu calorie labels, suggests a continued trend toward offering consumers lower-calorie choices.

Although calorie content of newly introduced items decreased overall after labeling, calorie content of new items increased at fast casual restaurants by 180 calories. The calorie content of removed items at fast casual chains, however, also increased after labeling by a similar amount (178 calories), which could offset the introduction of higher-calorie items. Continued monitoring of fast casual restaurants is warranted, given that these chains account for the majority of restaurants in most US counties.^30^

Analyses revealed a long-term decrease (−17 calories/year) in the calorie content of items removed from restaurant menus that began before labeling implementation; labeling was not associated with additional changes independent of this secular trend. This finding contrasts with a prior study finding that chain restaurants were eliminating high-calorie items from their menus prior to nationwide calorie labeling.^11^ Our study included a slightly different sample of chains and used data from 2012 to 2019, while the previous study used data from 2012 to 2015, perhaps explaining the discrepant findings. If the trend of removing lower-calorie items persists, it could offset potential benefits associated with the introduction of lower-calorie options.

Analyses of all menu items (ie, including continuously available items, new items, removed items, and all other items) found no changes in items’ calorie content after labeling. The lack of meaningful change in overall item calorie content suggests that labeling-induced product reformulation might take time to be reflected in menus as a whole and ultimately might not reduce consumption of higher-calorie foods at restaurants in the absence of other interventions. Policy makers could explore additional interventions that target both supply and demand, such as warning labels,^31,32,33,34^ healthy default options,^35,36^ reductions in portion size,^37^ and sweetened beverage taxes.^38,39^

### Limitations

This study has several limitations. First, we examined only top chain restaurants by sales volume, and our results may not be generalizable to restaurants that have lower sales volume but enough locations (≥20) to be subject to labeling requirements. Second, we could not examine a control group because all large chain restaurants were required to label their menus nationwide at the same time and only 2 failed to comply.^18^ The data also did not contain sufficient time points to conduct an interrupted time series analysis^40^; however, we adjusted for time trends in calorie content. Third, some items had missing calorie data. Although the rate of missingness was similar in the prelabeling and postlabeling periods overall, some subcategories (eg, fast-food restaurants) had a higher rate of missingness in 1 period, and we cannot rule out that missingness was associated with calorie content. In addition, some subcategories (eg, beverages, items at coffee restaurants) had a higher rate of missingness, and the results for these categories should be interpreted with more caution. Fourth, we were unable to examine changes in some popular restaurant chains that implemented labels before 2013 (eg, McDonald’s and Starbucks) because annual nutrition data were not available in MenuStat prior to 2012. Fifth, we focused on supply-side responses to calorie labeling and did not examine calorie intake; additional research should investigate consumer responses to calorie labels after nationwide implementation.

## Conclusions

This cohort study of 59 of the largest US chain restaurants found that menu calorie labeling was not associated with changes in the calorie content of existing menu offerings but was associated with a sizable decrease in the calorie content of newly introduced items, which account for approximately one-fifth of the menu items offered by chains in any given year. The introduction of lower-calorie items may be associated with reductions in calories consumed from restaurants; however, the heterogeneity in the new items’ calorie content by restaurant type warrants attention. Given the relatively low cost of implementing calorie labels,^15^ our results suggest that the US should continue to implement this intervention while also exploring additional strategies for improving the nutritional quality of foods purchased from restaurants.

## Notes

Supplement. **eMethods.** Supplementary Methodological Details **eFigure.** Flow Chart of Restaurant Chains and Menu Items Included in Primary Analyses **eTable 1.** Definitions of Menu Item and Restaurant Types **eTable 2.** Count of Menu Item-by-Year Observations in MenuStat and Percent of Observations Missing Calorie Information, Overall and by Labeling Period, Menu Item Type, and Restaurant Type, and Year **eTable 3.** Count of Menu Item-by-Year Observations in MenuStat and Percent of Observations Missing Calorie Information Among All Menu Items, Continuously Available Items, Newly Introduced Items, and Dropped Items **eTable 4.** Composition of Menu Item Types and Restaurant Types Among Menu Items Analyzed in the Pre-labeling and Post-labeling Period **eTable 5.** Composition of Menu Item Types and Restaurant Types Among all Menu Items in the MenuStat Database Compared to Menu Items Included in the Analytic Sample **eTable 6.** Unique Number of Menu Items With Calorie Information Sold in Chain Restaurants, Overall and by Labeling Period, Menu Item Type, and Restaurant Type **eTable 7.** Adjusted Changes (95% CI) in Mean Nutrient Content of Newly Introduced Items After Implementation of Calorie Labeling **eTable 8.** Adjusted Changes (95% CI) in Calorie Content of Menu Items After Implementation of Calorie Labeling, Sensitivity Analyses
eReferences.
