# Supplementary material for: Changes in Calorie Content of Menu Items at Large Chain Restaurants After Implementation of Calorie Labels
Source: JAMA Netw Open. 2021 Dec 30;4(12):e2141353. doi: 10.1001/jamanetworkopen.2021.41353 (PMC8719240; doi:10.1001/jamanetworkopen.2021.41353)
Supplement: Supplement. — eMethods. Supplementary Methodological Details eFigure. Flow Chart of Restaurant Chains and Menu Items Included in Primary Analyses eTable 1. Definitions of Menu Item and Restaurant Types eTable 2. Count of Menu Item-by-Year Observations in MenuStat and Percent of Observations Missing Calorie Information, Overall and by Labeling Period, Menu Item Type, and Restaurant Type, and Year eTable 3. Count of Menu Item-by-Year Observations in MenuStat and Percent of Observations Missing Calorie Information Among All Menu Items, Continuously Available Items, Newly Introduced Items, and Dropped Items eTable 4. Composition of Menu Item Types and Restaurant Types Among Menu Items Analyzed in the Pre-labeling and Post-labeling Period eTable 5. Composition of Menu Item Types and Restaurant Types Among all Menu Items in the MenuStat Database Compared to Menu Items Included in the Analytic Sample eTable 6. Unique Number of Menu Items With Calorie Information Sold in Chain Restaurants, Overall and by Labeling Period, Menu Item Type, and Restaurant Type eTable 7. Adjusted Changes (95% CI) in Mean Nutrient Content of Newly Introduced Items After Implementation of Calorie Labeling eTable 8. Adjusted Changes (95% CI) in Calorie Content of Menu Items After Implementation of Calorie Labeling, Sensitivity Analyses eReferences. [file jamanetwopen-e2141353-s001.pdf]

Supplementary Online Content

Grummon AH, Petimar J, Soto MJ, et al. Changes in calorie content of menu items at large chain restaurants after implementation of calorie labels. *JAMA Netw Open.* 2021;4(12):e2141353. doi:10.1001/jamanetworkopen.2021.41353

eMethods. Supplementary Methodological Details
eFigure. Flow Chart of Restaurant Chains and Menu Items Included in Primary Analyses
eTable 1. Definitions of Menu Item and Restaurant Types
eTable 2. Count of Menu Item-by-Year Observations in MenuStat and Percent of Observations Missing Calorie Information, Overall and by Labeling Period, Menu Item Type, and Restaurant Type, and Year
eTable 3. Count of Menu Item-by-Year Observations in MenuStat and Percent of Observations Missing Calorie Information Among All Menu Items, Continuously Available Items, Newly Introduced Items, and Dropped Items
eTable 4. Composition of Menu Item Types and Restaurant Types Among Menu Items Analyzed in the Pre-labeling and Post-labeling Period
eTable 5. Composition of Menu Item Types and Restaurant Types Among all Menu Items in the MenuStat Database Compared to Menu Items Included in the Analytic Sample
eTable 6. Unique Number of Menu Items With Calorie Information Sold in Chain Restaurants, Overall and by Labeling Period, Menu Item Type, and Restaurant Type
eTable 7. Adjusted Changes (95% CI) in Mean Nutrient Content of Newly Introduced Items After Implementation of Calorie Labeling
eTable 8. Adjusted Changes (95% CI) in Calorie Content of Menu Items After Implementation of Calorie Labeling, Sensitivity Analyses
eReferences.

This supplementary material has been provided by the authors to give readers additional information about their work.

© 2021 Grummon AH et al. *JAMA Network Open.*

eMethods. Supplementary Methodological Details

Data

The New York City Department of Health and Mental Hygiene (NYC DOHMH) did not release a MenuStat database in 2019. We therefore created our own database with nutrition information of items offered at the top revenue-generating restaurants in 2019 with guidance from the NYC DOHMH. This data capture was completed in January 2019 to be consistent with previous years of MenuStat's data collection.

For each restaurant listed in 2018 MenuStat, we took screenshots of menu information (i.e., item names and descriptions), photos, and all available nutrition information from the restaurant's U.S. website. We also downloaded PDFs of menus, if available, for each restaurant. For items that were customizable, we captured nutrition information for the default build only. If available, we also collected information on items' food category, serving size, and other descriptive variables (e.g., kids' meals or regional offerings). We entered all items into a dataset and assigned them the same MenuStat ID as in previous years if the item was offered previously. We did this by matching items on keywords in the item name or description. New items were assigned a unique MenuStat ID. Initial data entry was completed by research assistants and double-checked by other members of the research staff.

Analyses

As described in the main text, primary analyses estimated the association of calorie labeling implementation with mean calorie content of menu items using a pre-post design, adjusting for potential secular trends. All analyses regressed calories per item on labeling period (pre vs. post) and years since study start in 2012. More specifically, analyses took the general form:

$$Calories_{i,s,t} = \beta_0 + \beta_1 post_{s,t} + \beta_2 year_t$$

where $Calories_{i,s,t}$ denotes calories in menu item *i* at restaurant chain *s* in year *t*; *post* is an indicator for the post-labeling period for restaurant *s*, and *year* is a continuous variable indicating years since 2012. The coefficient β_1 indicates change in calorie content after labeling, adjusting for existing time trends. For analyses of all menu items, we included item-level random effects to account for repeated observations within items. For analyses of continuously available items, we accounted for repeated observations using item-level fixed effects, which control for time-invariant unobservable item characteristics. Analyses of newly introduced and dropped items, which did not contain repeated observations within items, included chain-level fixed effects. All analyses estimated robust standard errors clustered at the chain-level. Analyses of continuously available, newly introduced, and dropped items used wild cluster bootstrapping with 999 repetitions to adjust standard errors for the small number of clusters (i.e., restaurant chains);[1,2] to our knowledge, this method cannot be applied to analyses including random effects.[2]

© 2021 Grummon AH et al. *JAMA Network Open.*

eFigure. Flow Chart of Restaurant Chains and Menu Items Included in Primary Analyses

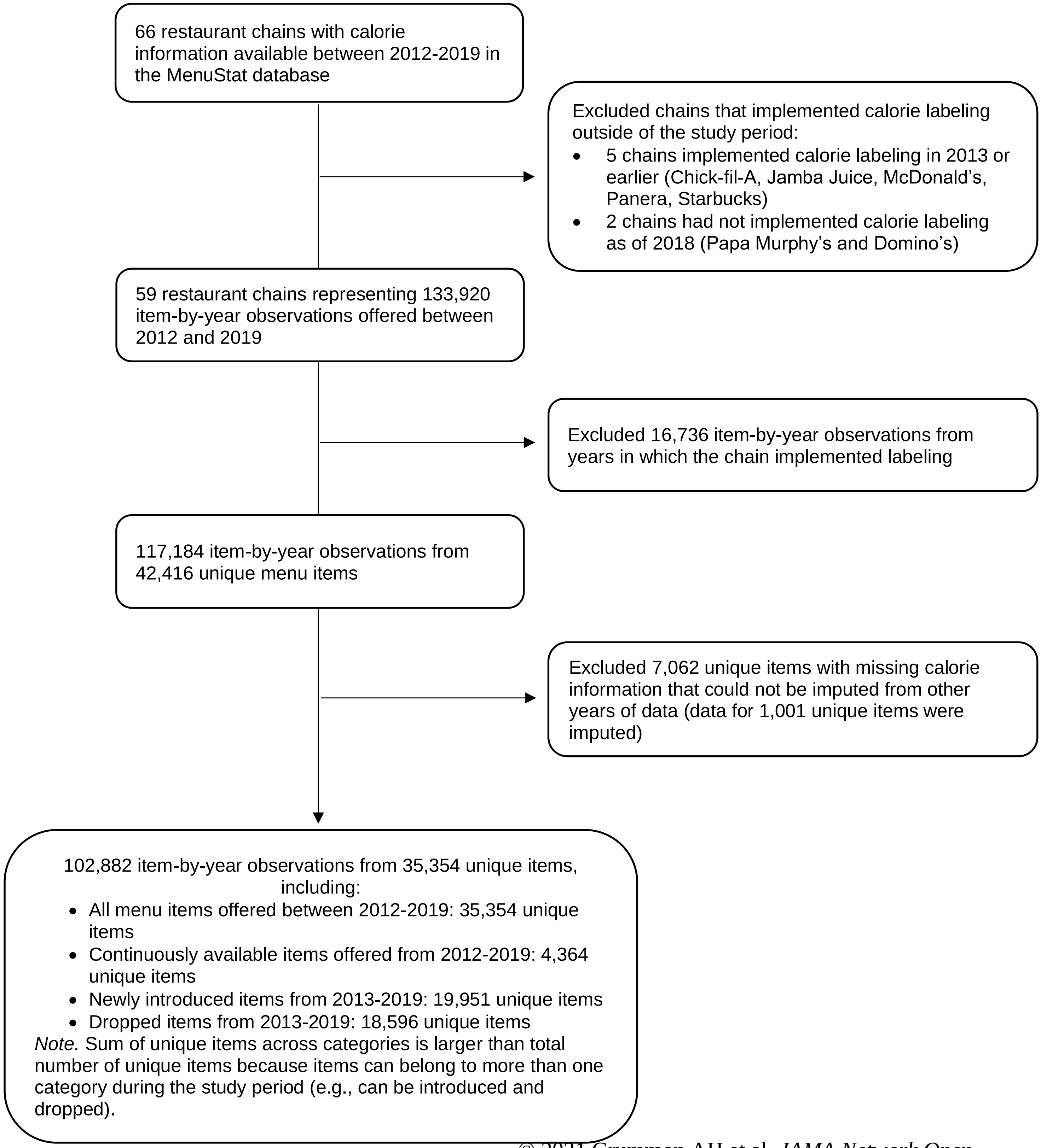

© 2021 Grummon AH et al. *JAMA Network Open.*

eTable 1. Definitions of Menu Item and Restaurant Types

Category	Description
**Menu item types**[a]	
Foods	All food categories below except for toppings and ingredients
Entrees	Burgers, pizza, salad, sandwiches, soups, and other entrees such as burritos, chicken, hot dogs, omelets, pancakes, pasta, seafood, steak, and tacos.
Appetizers and sides	Items sold as appetizers and sides or baked goods, including bagels, beans, bread, coleslaw, French fries and other fried potatoes, fruit, mozzarella sticks, onion rings, potatoes, rice, side salads, vegetables, wings
Desserts	Brownies, cakes, cheesecakes, cookies, donuts, fudge, ice cream, mousse, pie, sundaes
Toppings and ingredients	Toppings such as cheese, lettuce, onions, tomatoes; condiments and dips such as ketchup, mustard, mayonnaise, barbeque sauce, relish, sour cream; salad dressings; individual ingredients for build-your-own meal items including cold cuts, bread, bacon, pepperoni, pizza crust, tortillas; extra expresso shots; syrups and sweeteners; sauces such as cocktail sauce, marinara sauce, gravy, hot sauce
Beverages	Regular and diet sodas, fruit-flavored drinks, milkshakes, milk, lemonade, coffee, tea, iced tea, water, juice, alcohol, hot chocolate
**Restaurant types**[b]	
Fast food	Restaurants with no table service and meeting fewer than two of the criteria (listed below) for fast casual restaurants
Fast casual	Restaurants that had at least two of the following four criteria: 1) non-disposable utensils, 2) onsite food preparation, 3) no table service, and 4) commitment to higher-quality or fresh ingredients or sustainability
Full-service	Restaurants that self-identified on their website as full-service or that offer table service
Coffee	Restaurants that have coffee as the primary menu item or specialty based on the content of the restaurant's website

[a]Menu items were classified by MenuStat.[3]
[b]Restaurants were classified following Bleich et al.[4]

© 2021 Grummon AH et al. *JAMA Network Open.*

eTable 2. Count of Menu Item-by-Year Observations in MenuStat and Percent of Observations Missing Calorie Information, Overall and by Labeling Period, Menu Item Type, and Restaurant Type, and Year

	Total item-by-year observations (% of observations with missing calorie information)										
	**All item-year observations**	**Pre-label**	**Post-label**	**2012**	**2013**	**2014**	**2015**	**2016**	**2017**	**2018**	**2019**
**All menu items**	117,184 (12%)	80,979 (12%)	36,205 (14%)	11,671 (6%)	15,249 (8%)	16,052 (11%)	15,264 (14%)	20,018 (17%)	2,725 (12%)	17,256 (19%)	18,949 (9%)
**By menu item type**											
Food items[a]	60,905 (6%)	42,564 (5%)	18,341 (11%)	6,444 (1%)	8,332 (2%)	8,266 (4%)	7,789 (6%)	9,991 (8%)	1,742 (7%)	8,020 (11%)	10,321 (10%)
Entrees[b]	40,077 (7%)	27,799 (6%)	12,278 (11%)	4,000 (2%)	5,601 (3%)	5,483 (5%)	5,085 (8%)	6,507 (9%)	1,123 (8%)	5,389 (12%)	6,889 (11%)
Burgers	4,073 (7%)	2,864 (6%)	1,209 (9%)	432 (3%)	585 (2%)	542 (6%)	558 (5%)	671 (12%)	76 (4%)	540 (9%)	669 (9%)
Entrees	15,763 (9%)	11,131 (7%)	4,632 (15%)	1,572 (2%)	2,065 (4%)	2,085 (6%)	2,097 (10%)	2,614 (11%)	698 (10%)	1,799 (17%)	2,833 (13%)
Pizza	3,737 (2%)	1,883 (1%)	1,854 (2%)	345 (0%)	299 (0%)	382 (1%)	366 (1%)	475 (2%)	16 (0%)	1,014 (2%)	840 (2%)
Salad	3,339 (4%)	2,409 (3%)	930 (7%)	341 (1%)	488 (0%)	455 (2%)	466 (5%)	569 (4%)	90 (3%)	408 (7%)	522 (7%)
Sandwiches	11,103 (8%)	7,942 (6%)	3,161 (14%)	1,067 (3%)	1,820 (4%)	1,690 (5%)	1,307 (8%)	1,875 (9%)	183 (8%)	1,413 (15%)	1,748 (13%)
Soup	2,062 (2%)	1,570 (1%)	492 (4%)	243 (0%)	344 (1%)	329 (1%)	291 (1%)	303 (3%)	60 (2%)	215 (5%)	277 (4%)
Appetizers and sides[c]	12,885 (3%)	9,009 (2%)	3,876 (7%)	1,455 (1%)	1,640 (1%)	1,673 (1%)	1,679 (2%)	2,149 (3%)	413 (5%)	1,635 (5%)	2,241 (7%)
Appetizers and sides	7,625 (4%)	5,237 (2%)	2,388 (6%)	849 (1%)	941 (1%)	949 (2%)	969 (3%)	1,250 (4%)	279 (5%)	967 (7%)	1,421 (5%)
Baked goods	3,400 (3%)	2,432 (1%)	968 (9%)	386 (0%)	449 (0%)	456 (0%)	441 (1%)	621 (1%)	79 (8%)	429 (3%)	539 (14%)

© 2021 Grummon AH et al. *JAMA Network Open.*

Fried potatoes	1,860 (2%)	1,340 (0%)	520 (4%)	220 (0%)	250 (0%)	268 (0%)	269 (0%)	278 (1%)	55 (2%)	239 (3%)	281 (5%)
Desserts	7,943 (7%)	5,756 (4%)	2,187 (14%)	989 (0%)	1,091 (0%)	1,110 (1%)	1,025 (2%)	1,335 (15%)	206 (3%)	996 (18%)	1,191 (11%)
Toppings and ingredients	16,315 (3%)	11,500 (2%)	4,815 (3%)	1,707 (1%)	2,325 (1%)	2,290 (3%)	2,022 (2%)	2,755 (4%)	401 (5%)	2,434 (2%)	2,381 (4%)
Beverage items	39,964 (25%)	26,915 (26%)	13,049 (22%)	3,520 (16%)	4,592 (22%)	5,496 (25%)	5,453 (30%)	7,272 (33%)	582 (31%)	6,802 (34%)	6,247 (10%)
**By restaurant type**											
Fast food	52,940 (6%)	35,884 (4%)	17,056 (9%)	5,209 (1%)	7,228 (1%)	7,487 (4%)	6,705 (4%)	8,794 (8%)	461 (3%)	8,234 (8%)	8,822 (10%)
Fast casual	8,083 (8%)	5,511 (7%)	2,572 (11%)	822 (3%)	1,019 (6%)	1,119 (5%)	1,073 (6%)	1,315 (10%)	163 (31%)	1,063 (12%)	1,509 (10%)
Full Service	40,465 (11%)	29,014 (12%)	11,451 (8%)	4,568 (10%)	5,273 (10%)	5,501 (11%)	5,148 (14%)	6,652 (14%)	1,872 (13%)	4,743 (10%)	6,708 (7%)
Coffee	15,696 (40%)	10,570 (39%)	5,126 (42%)	1,072 (11%)	1,729 (31%)	1,945 (41%)	2,338 (47%)	3,257 (49%)	229 (8%)	3,216 (61%)	1,910 (10%)

Note. Data are from 35,354 menu items with calorie information offered at 59 restaurants in the MenuStat database from 2012 to 2019.
[a]Includes all food categories except toppings and ingredients.
[b]Includes burgers, entrees, pizza, salad, sandwiches, and soup.
[c]Includes appetizers and sides, baked goods, and fried potatoes.

© 2021 Grummon AH et al. *JAMA Network Open.*

eTable 3. Count of Menu Item-by-Year Observations in MenuStat and Percent of Observations Missing Calorie Information Among All Menu Items, Continuously Available Items, Newly Introduced Items, and Dropped Items

**Outcome**	**All observations**	**Pre-labeling**	**Post-labeling**
All menu items, N	117,184	80,979	36,205
% Missing	12%	12%	14%
Continuously available items, N	30,548	22,719	7,829
% Missing[a]	0%	0%	0%
Newly introduced items, N	26,075	16,553	9,522
% Missing	23%	25%	20%
Dropped items, N	24,339	14,406	9,933
% Missing	24%	17%	33%

[a]No continuously available items were missing calorie information because these items, by definition, had to have calorie information available in all years of MenuStat data.

© 2021 Grummon AH et al. *JAMA Network Open.*

eTable 4. Composition of Menu Item Types and Restaurant Types Among Menu Items Analyzed in the Pre-labeling and Post-labeling Period[a]

Item Characteristic	Pre-labeling % of observations	Post-labeling % of observations
**All menu items[b]**		
Menu item type		
Food[c]	53%	51%
Entrees[d]	65%	67%
Appetizers and sides[e]	21%	21%
Desserts	14%	12%
Toppings and ingredients	14%	13%
Beverages	33%	36%
Restaurant type		
Fast food	44%	47%
Fast casual	7%	7%
Full service	36%	32%
Coffee	13%	14%
**Continuously available items[f]**		
Menu item type		
Food[c]	62%	61%
Entrees[d]	60%	59%
Appetizers and sides[e]	24%	24%
Desserts	17%	17%
Toppings and ingredients	14%	13%
Beverages	24%	26%
Restaurant type		
Fast food	53%	58%
Fast casual	8%	8%
Full service	34%	28%
Coffee	6%	6%
**Newly introduced items[g]**		
Menu item type		
Food[c]	49%	42%
Entrees[d]	71%	73%
Appetizers and sides[e]	18%	18%
Desserts	11%	9%
Toppings and ingredients	12%	15%
Beverages	39%	43%
Restaurant type		
Fast food	38%	39%
Fast casual	6%	4%
Full service	35%	34%
Coffee	21%	23%
**Dropped items[h]**		
Menu item type		
Food[c]	56%	38%
Entrees[d]	75%	67%
Appetizers and sides[e]	14%	20%

© 2021 Grummon AH et al. *JAMA Network Open.*

Item Characteristic	Pre-labeling % of observations	Post-labeling % of observations
Desserts	11%	13%
Toppings and ingredients	12%	18%
Beverages	31%	44%
Restaurant type		
Fast food	40%	34%
Fast casual	6%	4%
Full service	39%	32%
Coffee	16%	30%

[a]Data are from 35,354 menu items with calorie information offered at 59 restaurants in the MenuStat database from 2012 to 2019. Table shows percent of item-by-year observations that were categorized as each menu item type and each restaurant type. Within categories, percentages may not sum to 100% due to rounding.
[b]Offered on any menu between 2012 and 2019.
[c]Includes all food categories except toppings and ingredients.
[d]Includes burgers, entrees, pizza, salad, sandwiches, and soup.
[e]Includes appetizers and sides, baked good, and fried potatoes.
[f]Items with the same or nearly identical name and description offered by the chain every year from 2012 to 2019 with calorie information available in all years.
[g]Not offered in 2012 and introduced after 2012.
[h]Offered in 2012 to 2018 and dropped between 2013 and 2019.

© 2021 Grummon AH et al. *JAMA Network Open.*

eTable 5. Composition of Menu Item Types and Restaurant Types Among all Menu Items in the MenuStat Database Compared to Menu Items Included in the Analytic Sample

Item Characteristic	Overall sample % of observations	Analytic sample % of observations
**All menu items**[b]		
Menu item type		
Food[c]	52%	55%
Entrees[d]	66%	65%
Appetizers and sides[e]	21%	22%
Desserts	13%	13%
Toppings and ingredients	14%	15%
Beverages	34%	29%
Restaurant type		
Fast food	45%	49%
Fast casual	7%	7%
Full service	35%	35%
Coffee	13%	9%
**Continuously available items**[f]		
Menu item type		
Food[c]	62%	62%
Entrees[d]	60%	60%
Appetizers and sides[e]	24%	24%
Desserts	17%	17%
Toppings and ingredients	14%	14%
Beverages	25%	25%
Restaurant type		
Fast food	54%	54%
Fast casual	8%	8%
Full service	32%	32%
Coffee	6%	6%
**Newly introduced items**[g]		
Menu item type		
Food[c]	46%	52%
Entrees[d]	72%	72%
Appetizers and sides[e]	18%	19%
Desserts	11%	10%
Toppings and ingredients	13%	17%
Beverages	40%	31%
Restaurant type		
Fast food	39%	45%
Fast casual	5%	6%
Full service	34%	37%
Coffee	22%	13%
**Dropped items**[h]		
Menu item type		
Food[c]	49%	56%
Entrees[d]	72%	73%

© 2021 Grummon AH et al. *JAMA Network Open.*

Item Characteristic	Overall sample % of observations	Analytic sample % of observations
Appetizers and sides[e]	16%	17%
Desserts	12%	11%
Toppings and ingredients	15%	18%
Beverages	37%	26%
Restaurant type		
Fast food	37%	44%
Fast casual	5%	5%
Full service	36%	39%
Coffee	22%	12%

[a]Data are from 35,354 menu items with calorie information offered at 59 restaurants in the MenuStat database from 2012 to 2019. Table shows percent of item-by-year observations that were categorized as each menu item type and each restaurant type. Within categories, percentages may not sum to 100% due to rounding.
[b]Offered on any menu between 2012 and 2019.
[c]Includes all food categories except toppings and ingredients.
[d]Includes burgers, entrees, pizza, salad, sandwiches, and soup.
[e]Includes appetizers and sides, baked good, and fried potatoes.
[f]Items with the same or nearly identical name and description offered by the chain every year from 2012 to 2019 with calorie information available in all years.
[g]Not offered in 2012 and introduced after 2012.
[h]Offered in 2012 to 2018 and dropped between 2013 and 2019.

© 2021 Grummon AH et al. *JAMA Network Open.*

eTable 6. Unique Number of Menu Items With Calorie Information Sold in Chain Restaurants, Overall and by Labeling Period, Menu Item Type, and Restaurant Type[a]

	All items offered from 2012 through 2019[b]		Offered every year from 2012 through 2019[c]		Newly introduced in 2013 through 2019[d]		Dropped in 2013 through 2019[e]	
**Category**	**Pre-label**	**Post-label**	**Pre-label**	**Post-label**	**Pre-label**	**Post-label**	**Pre-label**	**Post-label**
**All menu items**	25,620	21,359	4,364	4,364	12,334	7,617	11,893	6,703
**By menu item type**								
Food[f]	14,876	11,039	2,694	2,694	6,991	3,376	7,480	2,934
Entrees[g]	10,162	7,357	1,605	1,605	4,951	2,485	5,501	2,067
Appetizers and sides[h]	2,952	2,479	639	639	1,332	609	1,109	635
Desserts	1,762	1,203	450	450	708	282	870	232
Toppings and ingredients	3,961	3,516	595	595	1,874	1,431	1,717	1,685
Beverages	6,783	6,804	1,075	1,075	3,469	2,810	2,696	2,084
**By restaurant type**								
Fast food	12,007	9,769	2,350	2,350	5,615	3,306	5,387	2,747
Fast casual	1,756	1,541	353	353	742	358	714	307
Full service	9,320	7,770	1,414	1,414	4,529	2,879	4,584	2,626
Coffee	2,537	2,279	247	247	1,448	1,074	1,208	1,023

[a]Data are from 35,354 menu items with calorie information offered at 59 restaurants in the MenuStat database from 2012 to 2019.
[b]Offered on any menu between 2012 and 2019.
[c]Items with the same or nearly identical name and description offered by the chain every year from 2012 to 2019 with calorie information available in all years.
[d]Not offered in 2012 and introduced after 2012.
[e]Offered in 2012 to 2018 and dropped between 2013 and 2019.
[f]Includes all food categories except toppings and ingredients.
[g]Includes burgers, entrees, pizza, salad, sandwiches, and soup.
[h]Includes appetizers and sides, baked good, and fried potatoes.

© 2021 Grummon AH et al. *JAMA Network Open.*

eTable 7. Adjusted Changes (95% CI) in Mean Nutrient Content of Newly Introduced Items After Implementation of Calorie Labeling[a]

	**Saturated fat, g**			**Unsaturated fat, g**			**Sugar, g**			**Non-sugar carbohydrates excluding fiber, g**			**Protein, g**		
**Category**	**B**	**95% CI**	**Adj. *p***	**B**	**95% CI**	**Adj. *p***	**B**	**95% CI**	**Adj. *p***	**B**	**95% CI**	**Adj. *p***	**B**	**95% CI**	**Adj. *p***
**All menu items**	-2.2	(-4.5 to 0.03)	0.053	**-5.4**	**(-10.0 to -1.3)**	**0.007**	1.4	(-10.4 to 13.1)	0.808	**-4.3**	**(-9.0 to -0.1)**	**0.047**	**-6.9**	**(-13.5 to -1.3)**	**0.008**
**By menu item type**															
Food[b]	-0.1	(-2.6 to 2.3)	0.967	-2.8	(-9.8 to 2.9)	0.688	1.7	(-5.6 to 10.0)	0.982	3.8	(-4.8 to 12.7)	0.422	-4.4	(-14.0 to 3.2)	0.566
Entrees[c]	0.2	(-1.7 to 1.9)	0.967	-0.7	(-5.4 to 3.9)	0.842	-2.1	(-5.9 to 1.4)	0.742	4.1	(-5.9 to 13.7)	0.422	-1.5	(-6.9 to 3.9)	0.566
Appetizers and sides[e]	-2.2	(-9.0 to 3.0)	0.967	-6.2	(-25.4 to 7.3)	0.842	0.5	(-8.3 to 7.5)	0.982	8.1	(-4.4 to 22.3)	0.378	-15.8	(-45.2 to 4.3)	0.566
Desserts	-2.5	(-10.3 to 5.4)	0.967	-4.3	(-9.5 to 1.1)	0.276	0.2	(-19.3 to 27.9)	0.982	-4.2	(-14.7 to 1.4)	0.378	-0.9	(-3.5 to 2.9)	0.566
Toppings and ingredients	-1.2	(-2.4 to 0.4)	0.757	-1.5	(-3.8 to 1.1)	0.394	-3.3	(-10.2 to 2.2)	0.742	-7.1	(-15.9 to 0.8)	0.378	-4.0	(-11.6 to 1.8)	0.566
Beverages	-0.5	(-5.5 to 2.5)	0.967	-1.5	(-3.7 to 0.3)	0.276	-12.6	(-60.5 to 15.2)	0.982	-1.5	(-4.5 to 1.0)	0.378	-1.1	(-5.2 to 0.9)	0.566
**By restaurant type**															
Fast food	-1.9	(-6.5 to 1.6)	0.333	**-8.1**	**(-18.6 to -0.7)**	**0.031**	3.1	(-23.1 to 28.3)	0.761	**-9.0**	**(-14.4 to -3.9)**	**0.001**	**-12.6**	**(-28.7 to -2.9)**	**0.008**
Fast casual	3.6	(0.1 to 6.7)	0.052	**8.8**	**(2.0 to 13.9)**	**0.031**	-13.5	(-56.4 to 5.8)	0.761	**19.5**	**(8.5 to 37.2)**	**<0.001**	11.5	(-2.5 to 18.8)	0.125
Full service	**-3.4**	**(-6.7 to -0.5)**	**0.044**	-5.4	(-12.6 to 0.8)	0.081	-1.1	(-7.4 to 7.5)	0.761	-1.8	(-14.0 to 7.0)	0.760	-4.7	(-12.2 to 2.3)	0.211
Coffee	**-2.1**	**(-4.7 to -0.6)**	**<0.001**	**-1.2**	**(-3.1 to -0.2)**	**<0.001**	1.2	(-23.9 to 15.1)	0.761	**-3.6**	**(-6.1 to -2.2)**	**<0.001**	0.3	(-3.4 to 2.3)	0.750

[a]Data are from menu items with information on saturated fat sample (*n*=19,260), unsaturated fat (*n*=19,194), sugar (*n*=18,125) non-sugar carbohydrates (*n*=17,520), and protein (*n*=19,272) offered at 59 restaurants in the MenuStat database from 2012 to 2019. Table presents unstandardized regression coefficients and 95% CIs for the estimated change in mean nutrient content from pre- to post- implementation of menu calorie labels, adjusted for year (continuous), following the methods specified in the main text and eMethods. Newly introduced items were defined as those not offered in 2012 and introduced after 2012.). P-values were adjusted within families of outcomes (i.e., all menu items [1 test], menu item categories [6], and restaurant type categories [4]) by controlling the false discovery rate at q=0.05 using Benjamini and Hochberg's linear step-up method.[27] **Bold** estimates are statistically significant at p<0.05.
[b]Includes all food categories except toppings and ingredients.
[c]Includes burgers, entrees, pizza, salad, sandwiches, and soup.
[d]Includes appetizers and sides, baked good, and fried potatoes.

© 2021 Grummon AH et al. *JAMA Network Open.*

eTable 8. Adjusted Changes (95% CI) in Calorie Content of Menu Items After Implementation of Calorie Labeling, Sensitivity Analyses[a]

	Model 1			Model 2			Model 3			Model 4			Model 5			Model 6			Model 7		
**Category**	**B**	**95% CI**	**Adj. *p***	**B**	**95% CI**	**Adj. *p***	**B**	**95% CI**	**Adj. *p***	**B**	**95% CI**	**Adj. *p***	**B**	**95% CI**	**Adj. *p***	**B**	**95% CI**	**Adj. *p***	**B**	**95% CI**	**Adj. *p***
**All menu items[b]**																					
Model estimates	-1.1	(-3.0 to 0.8)	0.252	-2.1	(-8.7 to 4.5)	0.528	-2.6	(-9.3 to 4.1)	0.444	-1.5	(-8.0 to 4.9)	0.640	1.6	(-3.4 to 6.5)	0.537	-2.5	(-9.2 to 4.2)	0.466	-	-	-
Observations		35,354			35,209			34,225			35,310			29,816			33,950				
**Continuously available items[c]**																					
Model estimates	-0.8	(-2.1 to 0.4)	0.184	-2.2	(-11.3 to 6.3)	0.184	-3.0	(-12.4 to 5.5)	0.184	-0.7	(-9.5 to 7.4)	0.184	4.0	(-2.4 to 10.5)	0.184	-3.5	(-12.5 to 5.2)	0.184	-	-	-
Observations		4,364			4,219			4,179			4,364			3,465			4,260				
**Newly introduced items[d]**																					
Model estimates	**-99.7**	**(-122.9 to - 76.5)**	**<0.001**	**-110.2**	**(-210.4 to - 19.1)**	**0.014**	**-106.9**	**(-204.0 to - 21.3)**	**0.011**	**-88.9**	**(-168.0 to - 14.9)**	**0.014**	**-110.1**	**(-219.8 to - 16.9)**	**0.019**	**-113.7**	**(-216.6 to - 19.1)**	**0.012**	-61.9	(-201.5 to 68.9)	0.434
Observations		19,951			19,749			19,407			19,071			17,451			18,913			13,515	
**Dropped items[e]**																					
Model estimates	-1.2	(-21.8 to 19.4)	0.909	1.5	(-80.7 to 86.0)	0.969	4.3	(-79.2 to 81.2)	0.926	15.5	(-65.2 to 100.9)	0.700	10.8	(-79.8 to 100.0)	0.791	6.4	(-76.3 to 92.4)	0.883	-	-	-
Observations		18,596			18,396			18,048			18,298			16,405			17,517				

[a]Data are from 35,354 menu items with calorie information offered at 59 restaurants in the MenuStat database from 2012 to 2019. Table presents unstandardized regression coefficients and 95% CIs for the estimated change in mean calorie content from pre- to post- implementation of menu calorie labels, adjusted for year (continuous), following the methods specified in the main text and eMethods. Sensitivity analyses differed from the primary analyses in the following ways: Model 1 examined outcomes at the median using quantile regression; Model 2 analyses outcomes without imputing calorie content for any items; Model 3 excluded four restaurants for which >50% of locations were subject to local calorie labeling regulations prior to 2012; Model 4 recoded the nine chains that had regionally (but not nationally) implemented calorie labels in 2017 per Cleveland et al.'s audit[5] as implementing calorie labeling in 2018 instead of 2017; Model 5 includes only chains that implemented labeling in 2017, excluding those that were not compliant until 2018; Model 6 excludes regionally-available menu items; and Model 7 excluded limited-run newly introduced items, defined as those that were available only in the year they were introduced and not any years after. Model 7 applies only to newly introduced items and was not run for other outcome groups. **Bold** estimates are statistically significant at $p<0.05$.
[b]Offered on any menu between 2012 and 2019.
[c]Items with the same or nearly identical name and description offered by the chain every year from 2012 to 2019 with calorie information available in all years.
[d]Not offered in 2012 and introduced after 2012.
[e]Offered in 2012 to 2018 and dropped between 2013 and 2019.

© 2021 Grummon AH et al. *JAMA Network Open.*

eReferences.

1. Roodman D, Nielsen MØ, MacKinnon JG, Webb MD. Fast and wild: Bootstrap inference in Stata using boottest. *The Stata Journal*. 2019;19(1):4-60.

2. Cameron AC, Gelbach JB, Miller DL. Bootstrap-based improvements for inference with clustered errors. *Rev Econ Stat*. 2008;90(3):414-427.

3. New York City Department of Health and Mental Hygiene. MenuStat. Published 2021. Accessed February 8, 2021. http://menustat.org/#/home

4. Bleich SN, Wolfson JA, Jarlenski MP, Block JP. Restaurants with calories displayed on menus had lower calorie counts compared to restaurants without such labels. *Health Aff*. 2015;34(11):1877-1884.

5. Cleveland LP, Simon D, Block JP. Compliance in 2017 with Federal Calorie Labeling in 90 chain restaurants and 10 retail food outlets prior to required implementation. *Am J Public Health*. 2018;108(8):1099-1102.

6. Benjamini Y, Hochberg Y. Controlling the false discovery rate: a practical and powerful approach to multiple testing. *Journal of the Royal statistical society: series B (Methodological)*. 1995;57(1):289-300.

© 2021 Grummon AH et al. *JAMA Network Open.*
